# *NOX5* is expressed aberrantly but not a critical pathogenetic gene in Hirschsprung disease

**DOI:** 10.1186/s12887-021-02611-5

**Published:** 2021-03-30

**Authors:** Jing Wang, Jun Xiao, Xinyao Meng, Xufeng Chu, Di Di Zhuansun, Bo Xiong, Jiexiong Feng

**Affiliations:** 1 Department of Pediatric Surgery, Tongji Hospital, Tongji Medical College, Huazhong University of Science and Technology, 1095 Jiefang Ave, Wuhan, 430030 China; 2 Department of Forensic Medicine, Tongji Medical College, Huazhong University of Science and Technology, Hangkong road, Baofeng street, Qiaokou district, Wuhan, 430030 China

**Keywords:** Hirschsprung disease, *NOX5*, Enteric nervous system, Zebrafish, Embryo

## Abstract

**Background:**

Hirschsprung disease (HSCR) is a congenital disorder characterized by the absence of intramural ganglion cells in the distal gastrointestinal tract (GI), which results in tonic contraction of the aganglionic gut segment and functional intestinal obstruction. Recent studies have suggested *NADPH oxidase 5* (*NOX5*) as a candidate risk gene for HSCR. In this study, we examined the function of *NOX5* to verify its role in the development of the enteric nervous system (ENS).

**Methods:**

HSCR tissue specimens (*n* = 10) were collected at the time of pull-through surgery and control specimens (*n* = 10) were obtained at the time of colostomy closure in patients. The *NOX5* expression in aganglionic and ganglionic segments of HSCR colon and normal colon were analyzed by immunohistochemistry (IHC), western blot and real-time quantitative PCR (qPCR). The gene expression levels and spatiotemporal expression spectrum of *NOX5* in different development stages of zebrafish embryo were determined using qPCR and in-situ hybridization (ISH). The enteric nervous system in *NOX5* Morpholino (MO) knockdown and wild type (WT) zebrafish embryo was analyzed by whole-mount immunofluorescence (IF). Intestinal transit assay was performed to analyze the gastrointestinal motility in *NOX5* knockdown and control larvae.

**Results:**

*NOX5* is strongly expressed in the ganglion cells in the proximal segment of HSCR colons and all segments of normal colons. Moreover, the expression of *NOX5* is markedly decreased in the aganglionic segment of HSCR colon compared to the ganglionic segment. In zebrafish, *NOX5* mRNA level is the highest in the one cell stage embryos and it is decreased overtime with the development of the embryos. Interestingly, the expression of *NOX5* appears to be enriched in the nervous system. However, the number of neurons in the GI tract and the GI motility were not affected upon *NOX5* knockdown.

**Conclusions:**

Our study shows that *NOX5* markedly decreased in the aganglionic segment of HSCR but didn’t involve in the ENS development of zebrafish. It implies that absence of intestinal ganglion cells may lead to down-regulation of *NOX5*.

**Supplementary Information:**

The online version contains supplementary material available at 10.1186/s12887-021-02611-5.

## Background

HSCR is caused by failures of the proliferation, differentiation or migration of enteric neural crest cells (ENCCs), resulting in aganglionosis of the distal part of the gastrointestinal tract [1]. According to the length of the aganglionic segment, it can be further classified as short segment HSCR, long segment HSCR, total colonic aganglionosis (TCA) and total intestinal aganglionosis [2]. The aganglionosis then leads to severe intestinal obstruction, which requires surgical removal of the aganglionic segment to treat [3].

HSCR is a highly heritable disorder (OMIM 142623). Familial and syndromic HSCR show a Mendelian pattern of inheritance. However, the etiology of the sporadic HSCR seems to be intricate, presenting a non-Mendelian type inheritance and involving many genetic and environmental factors [4]. So far, more than 20 HSCR susceptibility genes have been found to be associated with the development of ENS and most of them were involved in the RET (encoding a tyrosine kinase) and EDNRB signaling pathways [5, 6]. The RET/GDNF/GFRA1 signaling pathway and the Endothelin 3-Endothelin Receptor B signaling pathway are the most common known pathways involved in HSCR development. Besides, transcription factors like SOX10, PHOX2B, ZEB2 etc. are also playing important roles in the development of ENS [4]. Recent studies have also demonstrated the association between SEMA3 and HSCR, which was applicable to specific ethnic groups [7–9]. However, only approximately 30% of all HSCR patients carry mutations in these genes, suggesting that there must be many other genes participating in the etiology of HSCR.

In the last few years, lots of novel susceptibility genes and variants for HSCR were identify by genome-wide association studies (GWAS) [10, 11]. In a recent GWAS of HSCR, *NOX5* was identified as a susceptibility gene [12]. Furthermore, a follow-up association analysis between *NOX5* polymorphisms and risk of HSCR indicated that several hereditary variants in *NOX5* were significantly associated with HSCR susceptibility [13].

*NOX5*, which belongs to the NADPH oxidase family, is one of the major producers of reactive oxygen species (ROS) in mammalian cells [14]. NADPH oxidases are membrane proteins that generate superoxides, particularly ROS, which have been shown to be participated in various signaling cascades and cellular processes including proliferation, apoptosis and migration. Dysfunction of the NOX enzyme could lead to abnormal levels of ROS that may cause diseases [15]. Previous studies have demonstrated that *NOX5* is involved in various pathological conditions, including cancer, cardiovascular and atherosclerotic diseases [16–18]. However, no in vivo or in vitro study has provided immediate evidence that *NOX5* is required for the development or function of the ENS. In this study, we aimed to test whether loss of *NOX5* would lead to the disruption of the biological processes of enteric neurons using zebrafish models.

## Methods

### Tissue collection

This study complied with the Declaration of Helsinki and was approved by the Review Board of Ethics Committee of Tongji Hospital. Consent forms were sent to patients at the age between 0 and 5 years and signed by their legal custodians. Full-length resected bowel specimens obtained during pull-through operations for HSCR were collected from ten patients. Three of these patients had a history of preoperative HAEC. Resected tissues included aganglionic and ganglionic segments. Ganglionic segments were taken from the most proximal margin of the resected pull-through specimen while aganglionic segments were taken from the most distal margin of the resected specimen. Control group of specimens were obtained from imperforate anus patients after colostomy (*n* = 10). Tissue specimens were stored in three ways following collection. One portion of each specimen was fixed in formalin at room temperature, for paraffin embedding and immunochemistry. A second portion was snap frozen in a mold containing optimal cutting temperature medium and stored at − 80 °C for immunofluorescence. The remaining specimen was stored at − 80 °C for protein or total RNA extraction.

### Protein extraction and Western blot

Each protein sample of zebrafish was extracted from 30 embryos. The western blot was then performed as previously described [19]. The rabbit anti-*NOX5* antibody (Abcam, Cambridge, UK, ab191010) was used at a concentration of 1:1000. The rabbit anti-beta III Tubulin (Tuj1) antibody (Abcam, Cambridge, UK, ab18207) was used at a concentration of 1:1000. The HRP linked goat anti-rabbit secondary antibody (Abcam, Cambridge, UK) was used at 1:10,000 dilution. Beta actin (dilution 1:1000, Abcam, Cambridge, UK) was used as the loading control. The relative level of protein was determined by the normalized density of each band in the western blot using the ImageJ software.

### Immunohistochemistry

Sections (4 μm) on silane-coated slides (Muto Pure Chemicals Co., Ltd., Tokyo, Japan) were deparaffinized in xylene and dehydrated in solutions with decreasing concentrations of ethanol. After rehydration and blocking of endogenous peroxidase activity with 3% of hydrogen peroxide for 10 min, heat-induced epitope retrieval was performed for 20 min in 0.01 M citrate buffer (pH 6.0) in a pressure cooker. Primary antibody for *NOX5* was used at 1:100 and incubated for 30 min. After washing and incubation with EnVision™ for 30 min, color products were developed using the Liquid DAB+ as chromogen. The sections were counterstained with hematoxylin before dehydration and coverslipping. Slides processed without primary antibody were prepared as negative controls.

### Zebrafish housing/breeding

The zebrafish protocols were approved by the Institutional Animal Care and Use Committee at Tongji Hospital. The AB strain zebrafish were maintained according to standard procedures [20]. Embryos were raised in E3 medium at 28.5 °C and staged as previously described [21]. 0.003% N-phenylthiourea was added to E3 medium to inhibit melanization.

### Real-time quantitative PCR

Total RNA was isolated from 0.2, 6, 12, 24, 48, and 72hpf embryos and colon tissues with TRIzol reagent (Life Technologies, Carlsbad, CA). qPCR and data analysis were performed using LightCycler96 (Roche Diagnostics). Relative expression levels were calculated using β-actin as internal reference. The experiments were repeated three times with biological replicates. Zebrafish primers (*NOX5* primer: Forward, 5′- ATT CACGGCACT GAAACGGA-3′, Reverse, 5′-GGAGCTCCGCATGATT TACCT A-3′; β-actin primer: Forward, 5′-CGAGCTGTCTTCCCATCCA-3′, Reverse, 5′-TCACC AACGTAGCTG TCTTTCTG-3′). Human primers (Tuj1 primer: Forward, 5′- GGA AGAGGGCGAGATGTACG-3′, Reverse, 5′- GGGTTTAGACACTGCTGGCT-3′; β-actin primer: Forward, 5′- CCTTCCTGGGCATGGAGTC-3′, Reverse, 5′- TGA TCTTCATTGTGCTGGGTG-3′. *NOX5* primer: Forward, 5′-CCAGAAAGTGGCTG CTGAGA-3′, Reverse, 5′-AGCTTGGAGAGGTGAGGCTA-3).

### Whole-mount in situ hybridization

The 0.2, 6, 12, 48hpf Embryos (*n* = 15 for each phase) were collected and processed for whole-mount ISH as previously described [22].

A 735-bp fragment of the *NOX5* cDNA was amplified using the following primers: Forward: 5′-CGGAGGTCTCTGGATCATGC-3′, Reverse: 5′-ATGTGCAGCCACAA CGTTTC-3′. A T7 promoter was added to the reverse primer. The ISH probe was then generated by in vitro transcription using T7 RNA polymerase.

### Microinjection of morpholino antisense oligonucleotides

*NOX5* knockdown experiments using MO were carried out as previously described [23]. ATG morpholino antisense oligonucleotides targeting *NOX5* were designed and synthesized as follows: *NOX5*-MO 5′-CGGGTGTCATCATCCAGACTCAT-3′, a 5-nucleotide-mismatch morpholino was used as control: 5′- CGGcTGaCtTgATCCAcAC TCAT − 3′. Zebrafish embryos were injected with 5 ng of the MO at the one cell stage. The knockdown efficiency was validated using western blot.

### Whole-mount Immunofluorescent staining

Whole-mount Immunofluorescent staining with the anti-HuC/D antibody (A-21271, Life Technologies) was performed to examine the enteric neurons along the GI tract. The 5dpf embryos (*n* = 20) were collected and fixed with 4% PFA overnight. The embryos were washed with PBS. After incubation in blocking solution (2% goat serum, 2 mg/ml BSA in 1 x PBS) for 1 h at room temperature, embryos were incubated with the anti-HuC/D antibody (1500) in blocking solution overnight at 4 °C. After two washes in PBS for 10 min each time, embryos were incubated in the secondary antibody solution, 1: 1000 Alexa Fluor rabbit anti-mouse IgG (A11001, Life Technologies) in PBS, for 1 h at room temperature. Finally, the images were acquired using LSM 800 confocal microscope (Zeiss, Germany). The number of HuC/D-positive cells in the gut was then quantified using ImageJ. All of the experiments were repeated for three times.

### Zebrafish intestinal transit assay

The tracer was prepared by mixing 100 mg of egg yolk, 150 μL of yellow-green fluorescent 2.0-um polystyrene microspheres (Invitrogen, Carlsbad, CA, USA) and 50 μL of deionized water as previously described [24]. For 7dpf zebrafish larvae (*n* = 65 for Control, *n* = 75 for *NOX5*-Mo), approximately 2 mg of tracer powder was administered per Petri dish in the morning. After 3, 6, and 9 h, the larvae were anaesthetized by 0.2%. tricaine (Sigma, St Louis, MO, USA) and imaged using a fluorescent dissecting microscope (Axio Zoom.V16, Zeiss, Germany). For scoring the transit efficiency, the zebrafish intestine was artificially divided into four zones according to anatomical landmarks and the larvae was grouped based on the anterior extent of the tracer.

### Statistical analysis

The embryos were selected by Simple random sampling. Data were analyzed using the GraphPad Prism software package (version 5; GraphPad Software Inc., La Jolla, CA, USA) and are presented as the mean ± standard error of the mean. Differences between two groups were analyzed using an unpaired t-test with Welch’s correction. Analysis of variance (ANOVA) was used to compare data of more than two groups. Pearson’s chi-square tests were used to assess the difference between 7 dpf wild-type and *NOX5*-MO group transit profiles at different time points. The experiments and data analyzing were finished by different researchers. The analyst didn’t know the grouping scheme in advance.

## Results

### *NOX5* is located in the enteric neuron membrane

**Fig. 1:**
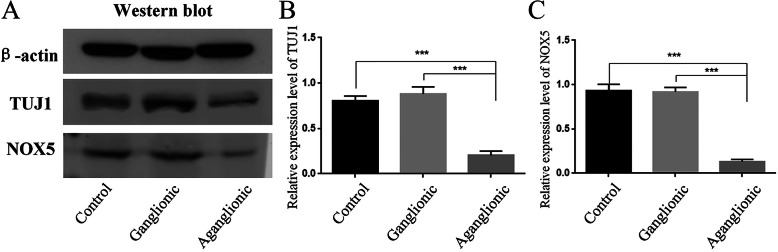
Western blotting revealed significantly increased protein expression levels of NOX5 and Tuj1 in the ganglionic HSCR specimens (*n* = 10) compared to aganglionic and normal control tissue (*n* = 10). Equal loading of electrophoresis gels was confirmed by ACTIN staining (*p* < 0.001 by one-way ANOVA)

**Fig. 2:**
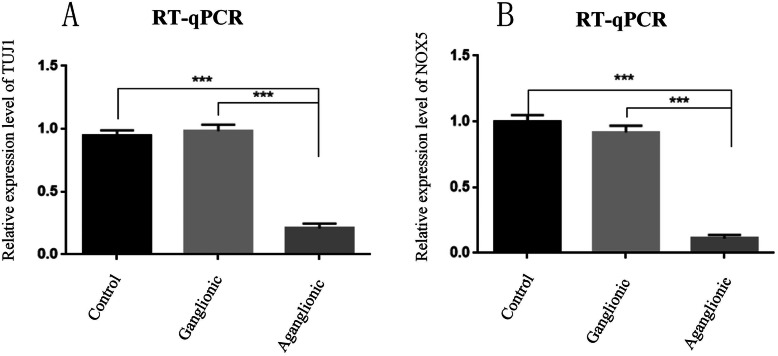
The qPCR results revealed significantly increased expression of NOX5 and Tuj1 in the ganglionic HSCR specimens (*n* = 10) compared to aganglionic and normal control tissue (*n* = 10). (*p* < 0.001 by one-way ANOVA)

**Fig. 3:**
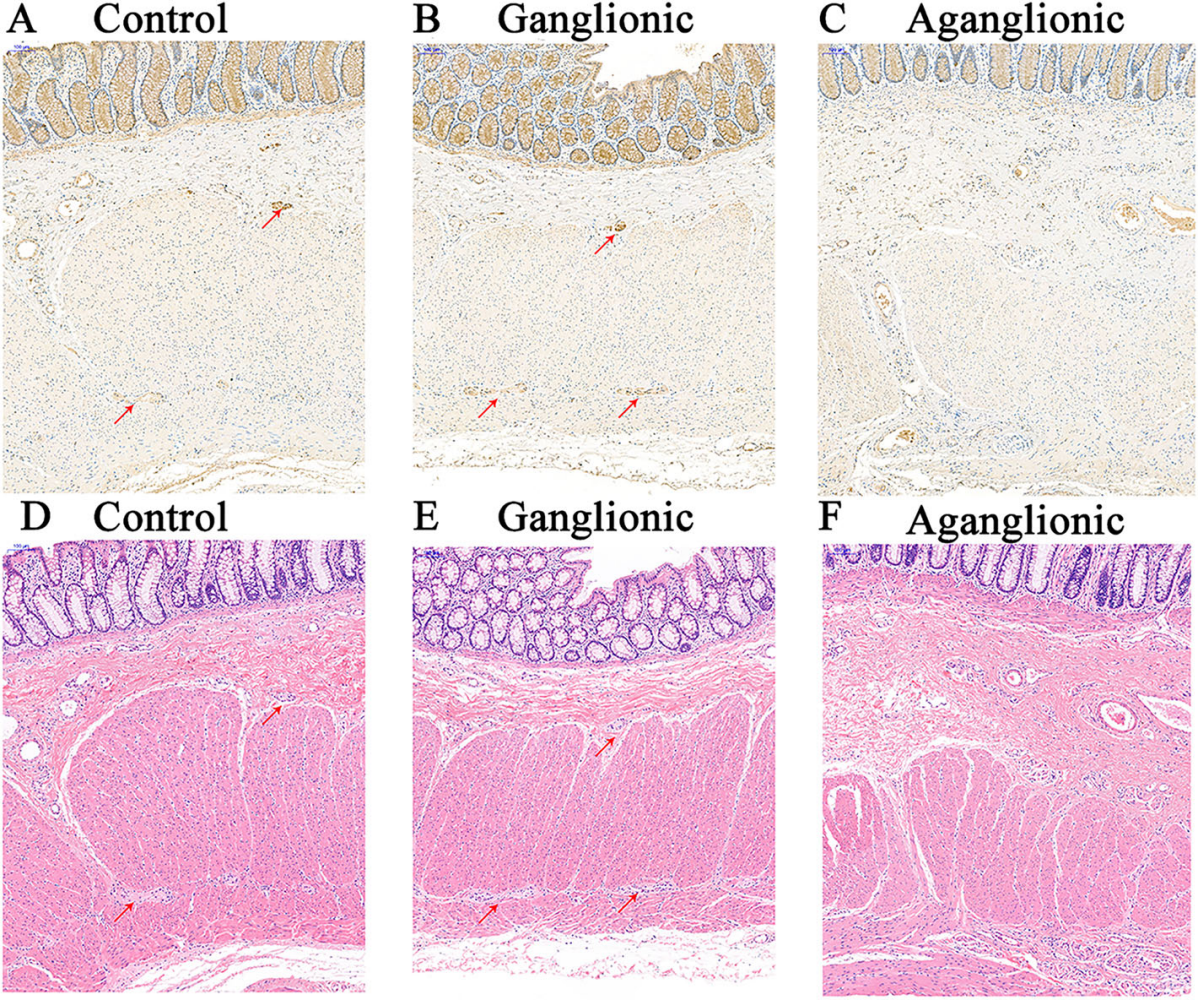
NOX5 antibodies positively stained ganglion cells (arrows) in the submucosal and myenteric plexus of the ganglionic (**a**, **d**, × 100) and normal colon (**c**, **f**, × 100) but not aganglionic colon (**b**, **e**, × 100). Each ISH section have a correspondent HE staining slide

We first performed western blots and qPCR of *NOX5* in the aganglionic segments and the ganglionic segments in 10 patients with HSCR and 10 normal colon samples. The results shown that *NOX5* is indeed expressed in the colon. The ganglionic segments in HSCR patients and imperforate anus patients had a similar expression level of NOX5. However, the expression level of *NOX5* in the aganglionic segments were markedly decreased (Figs. 1 and 2). Besides, the expression level of *NOX5* is similar to Tuj1, which is a neuron specific marker in ENS. Next, we performed IHC and HE staining to examine the expression profile of *NOX5* in the colon. *NOX5* expression was observed in the ganglion cells in the myenteric and submucosa plexus of both the HSCR and control colons. However, *NOX5* is hardly expressed in aganglionic segments (Fig. 3). Collectively, these data indicated that *NOX5* is highly expressed in the ganglionic neurons of the colon, suggesting that it may play certain roles in these cells.

### Expression pattern of *NOX5* in zebrafish embryos

**Fig. 4:**
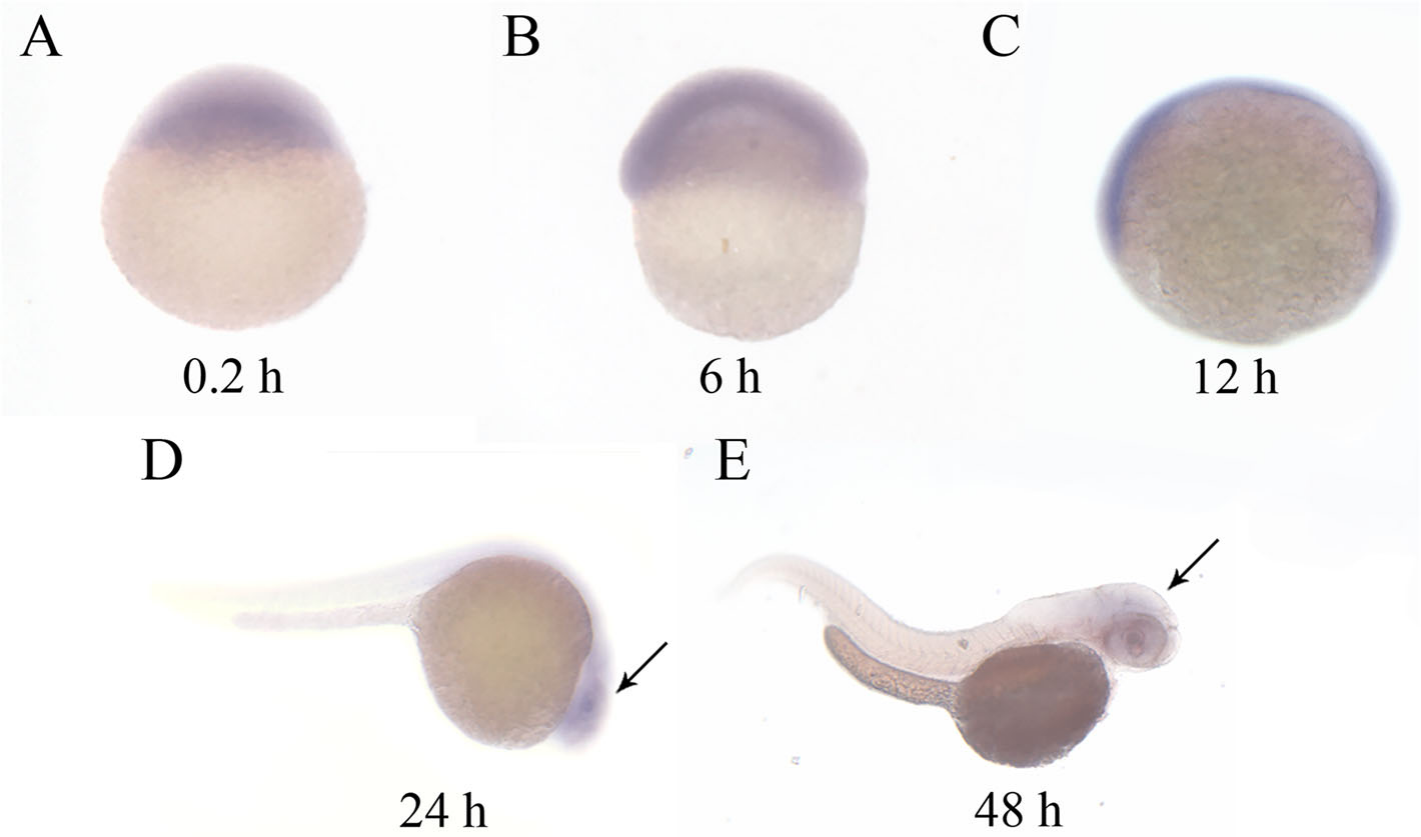
Broad NOX5 expression through the first 2 days of development. **a**-**e**: 0.2, 6, 12, 24, and 48 hpf. **d**-**e**: Lateral views of whole-mount ISH embryos probed with NOX5 antisense show a relative specific positive expression in central nervous system

**Fig. 5:**
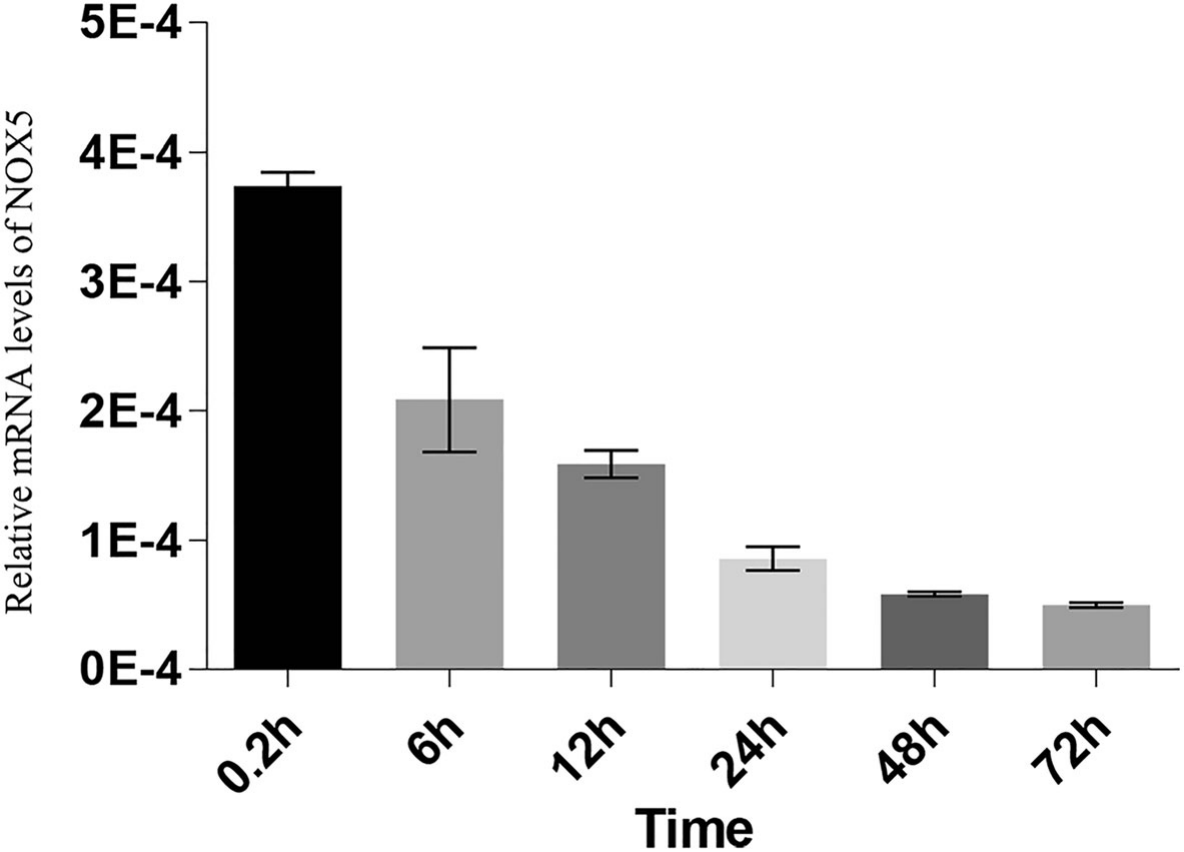
NOX5 expressed during early zebrafish development. Relative mRNA levels of NOX5 derived via quantitative PCR normalized to ACTIN. *N* = 6 for each data point

To analyze the function of *NOX5* in vivo, we chose to use zebrafish as a model system. We performed ISH to determine the *NOX5* expression pattern in different stages (0.2, 6, 12, 24, 48, 72hpf) of zebrafish embryos. *NOX5* started to express at 1-cell stage and is uniformly expressed pattern during the early stages of embryonic development (Fig. 4 a-c). After 24hpf, it is mainly expressed in the central nervous system, which is the origin of enteric neurons (Fig. 4 d-e). In addition, to qualify the relative level of *NOX5* in different stages, we performed qPCR analyses and revealed that *NOX5* expression is the highest in the one-cell stage and is decreased in the following stages until reaching plateau at around 48hpf to 72hpf (Fig. 5). Since the zebrafish enteric neurons are differentiated and migrated to their final location within the first 5 days of embryonic development, our data indicate that *NOX5* is indeed expressed during the period of enteric neuron development.

### *NOX5* is not required for zebrafish ENS development

**Fig. 6:**
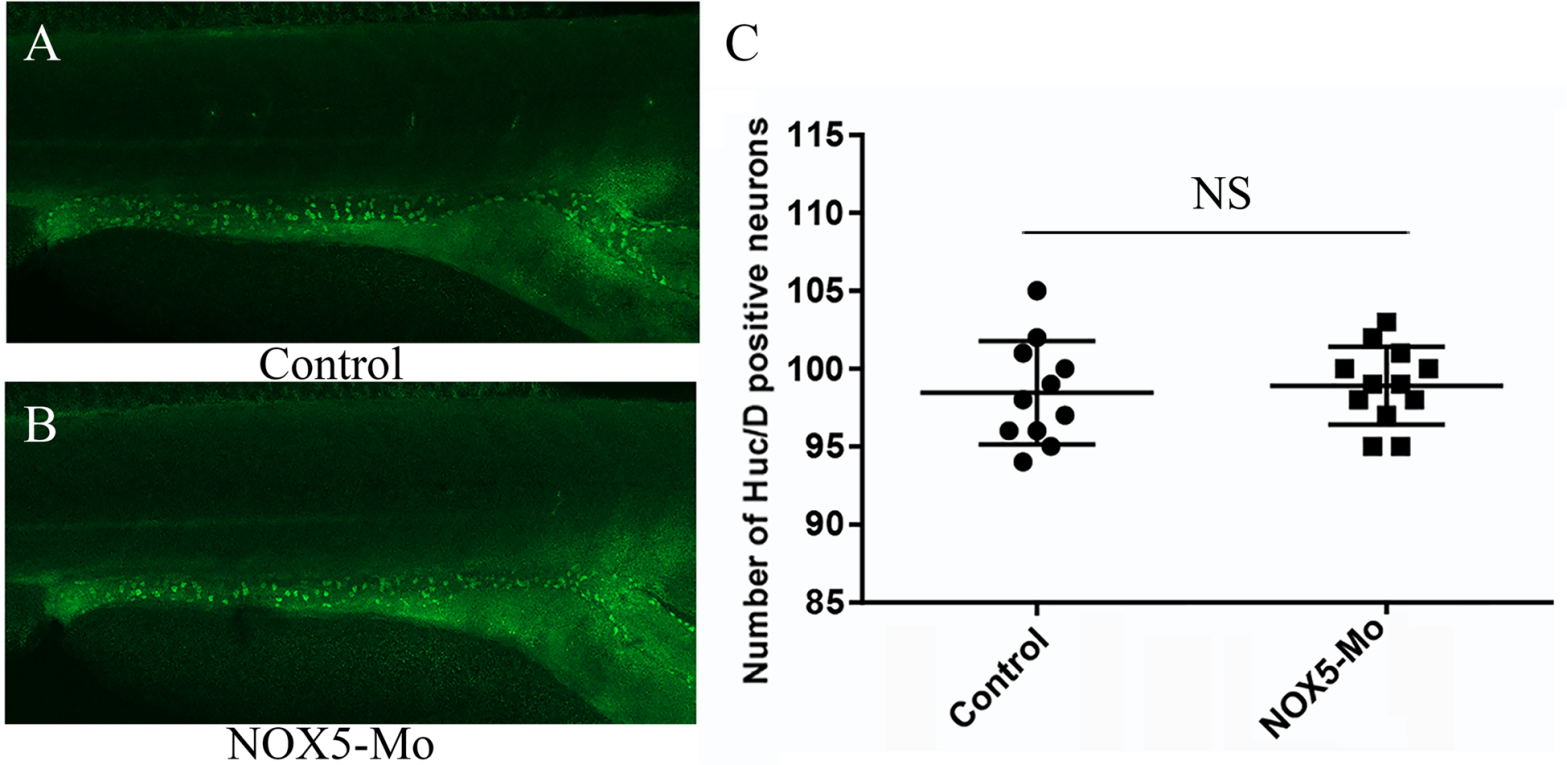
Immunostaining with HuC/D at 5dpf show no difference between control group (**a**) and NOX5-MO group (**b**). No statistical difference in HuC/D+ neuron numbers between two groups (**c**)

To test whether zebrafish ENS development require *NOX5*, we designed a MO to knockdown *NOX5* expression level. Upon injection of 5 ng *NOX5*-MO at one cell stage, the protein levels of *NOX5* were significantly reduced until 5dpf (Fig SF1). We then used HuC/D immunofluorescence staining to detect the enteric neurons in 5dpf embryos. The results showed that the number of enteric neurons in *NOX5* morphants are not significantly altered compare to controls (Fig. 6a-c). Moreover, the gut morphology was indistinguishable between the morphants and the controls. Thus, our data indicate that *NOX5* is dispensable for enteric neuron system development in zebrafish.

### GI transit function is not affected by *NOX5* knockdown

**Fig. 7:**
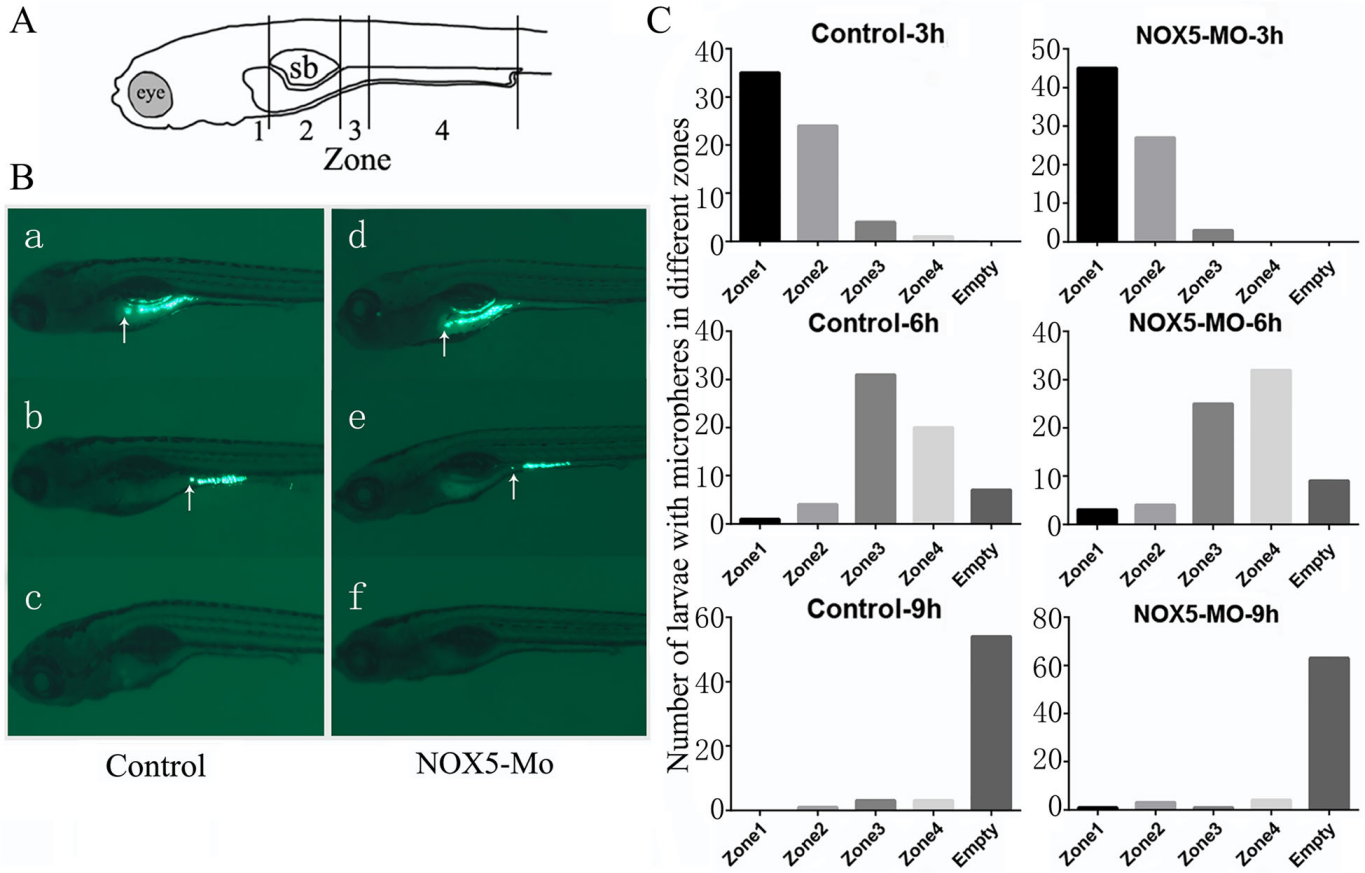
To evaluate intestinal transit at the population level, we categorized individuals within a population based on the location of tracer in the gastrointestinal tract. **a** Regions of the gut are divided to 4 zones based on anatomical landmarks. Zone 1 is the region of the intestinal bulb rostral to the swim bladder. Zone 2 is the region of the intestinal bulb ventral to the swim bladder. Zone 3 is the junction between the intestinal bulb and the mid intestine. Zone 4 consists of the mid- and posterior intestine. e, eye; sb, swim bladder. **b** Larvae were scored as zone 1, zone 2, zone 3, zone 4 and empty based on the anterior extent of tracer in the gastrointestinal tract (arrow). **c** Bar graphs representing the transit profile of a population of 7 dpf larvae at 3 h, 6 h, 9 h of transit time. Data are represented as the number of larvae classified into each zone category based on visual analysis of live larvae at the time specified. The difference of transit profiles of all time points between two groups show no statistically significant (*P* > 0.05)

We further tested whether *NOX5* is required for GI transit function by feeding larvae fluorescent microspheres in an emulsion of egg yolk and examining the microsphere transit through the GI at various time points. Upon feeding, both the control and *NOX5* knockdown group larvae were anaesthetized and photographed at 3, 6 and 9 h. For quantification, we divided the whole GI into four zones and calculated the number of larvae with the tracer in each zones (Fig. 7a). Representative images of the tracer location in controls and morphants at different time points are displayed in Fig. 7b. There is no significant difference of the tracer patterns between the two groups of embryos (Fig. 7c, 3 h, *p* = 0.32, 6 h, *p* = 0.49, 9 h, *P* = 0.59). These data suggested that *NOX5* is not required for ENS function or GI transit in zebrafish embryos.

## Discussion

HSCR is a highly heritable disorder [25]. Previously, a genome-wide association study (GWAS) with 123 sporadic HSCR patients and 432 unaffected controls identified *NOX5* as a new susceptibility gene for HSCR [12]. Furthermore, an association analysis between *NOX5* polymorphisms and risk of HSCR in 187 patients and 283 unaffected controls showed that the genetic variants in *NOX5* were significantly associated with HSCR susceptibility, particularly for the L-HSCR and TCA subtypes [13]. Encouraged by these findings, we embarked on elucidating *NOX5* function in ENS.

NADPH oxidases (*NOX*), comprising seven family members (*NOX1*-*NOX5* and dual oxidase 1 and 2), are the major producers of reactive oxygen species in mammalian cells. *NOX5* was first reported in 2001 based on a blast search using the C-terminus of gp91^phox^ as bait to identify novel transcripts [26]. The exact pathophysiological significance of *NOX5* remains unclear, but it seems to be important in the physiological regulation of sperm motility, vascular contraction and lymphocyte differentiation, and *NOX5* hyper activation has been implicated in cardiovascular disease, kidney injury and cancer. One of the distinguishing features of *NOX5* is the dependence on Ca^2+^ for its regulation [27]. Activation of *NOX5* in response to elevated Ca^2+^ is a multi-phased process [28]. The amount of Ca^2+^ required to activate *NOX5* fully is relatively high, and accordingly, additional systems involving regulatory proteins are operational that increase sensitivity to Ca^2+^, thereby facilitating ROS generation. Hence, *NOX5* can be activated directly by Ca^2+^ or indirectly by interacting with other proteins and kinases, such as Ca^2+^-bound calmodulin or PKC [29]. Intriguingly, *NOX5* is the first and only NADPH oxidase to be crystallized, providing opportunities to design specific *NOX5* inhibitors and activators, which is crucial for biomedical research and potentially for therapeutic utility [30].

We used zebrafish as the animal model to explore the function of *NOX5*. The result of spatiotemporal expression spectrum of *NOX5* in zebrafish embryo indicated that *NOX5* might play a role in the early development of zebrafish, which is similar to the previous study about the expression of NOX family in zebrafish [31]. However, after significant knockdown of the *NOX5* expression, there were no difference of HuC/D positive neuron numbers of the GI tract between the normal group and the *NOX5* knockdown group, indicated that the development of ENS in zebrafish do not require the *NOX5*. Besides, the results of GI transit assay suggested that the GI motility were not affected by the absence of *NOX5* protein either. Generally, GI motility is controlled by enteric neural crest cells that form the ENS and undergo extensive migration from the caudal hindbrain to colonize the total GI tract [32, 33]. The results of the in vivo study in zebrafish showed that the loss function of *NOX5* did not cause the absence of enteric neuron in zebrafish, which is the most important characteristic of HSCR.

Interestingly, our data of IHC showed that *NOX5* is indeed located on the enteric neuron membrane. qPCR, western blot showed that *NOX5* is strongly expressed in the myenteric ganglionic cell in the proximal and normal segment of HSCR colon and hardly expressed in the distal segment of colon with the absence of ganglionic cell. Recent researches have revealed that several aberrant gene expressions were involved in the pathological processes of HSCR, including UBR4 [34]. UBR4, a ubiquitin ligase protein, has been showed to be a novel HSCR gene [6]. It was required for neurogenesis and played an important role in myofiber hypertrophy [35, 36]. Therefore, NOX5 might be the downstream factor of UBR4. Downregulation of UBR4 expression might impact the development of ENS which probably reduce the expression of NOX5. On the one hand, abnormal distribution of ICCs had been observed in the aganglionic segment colon of HSCR patients [1]. What’ more, cell migration, contraction and proliferation cannot complete without Ca2^+^-dependent processes, and this is particularly pertinent to *NOX5*, because *NOX5* itself is regulated by Ca2^+^ [37]. Moreover, in bowel motility, Ca2^+^ is regulated by ICCs, which are essential to generate and propagate the electrical cyclical activity (slow waves) in the intestines. Therefore, we hypothesize that the aberrant expression of *NOX5* in aganglionic segment may occur due to the abnormal release of Ca2^+^. More importantly, our study demonstrated that *NOX5* expresses in the ganglionic cells specifically in colon tissue. *NOX5* may serve as a typical neuron marker for enteric ganglionic cell to determine the occurrence and development of HSCR. It is noteworthy that the disruption of the balance between Ca2^+^ and *NOX5* may lead to further deterioration of spasm in distal segment of HSCR patients. Potentially, abnormal release of Ca2^+^ may result in the decreased expression of *NOX5* through an unknown mechanism, which may further cause dysregulation of Ca2^+^ the concentration.

However, the association between *NOX5* and Ca2^+^ remain unclear and further studies are required to explain the decreased level of *NOX5* in the aganglionic segment colon of HSCR patients.

## Conclusions

Our study shows that *NOX5* markedly decreased in the aganglionic segment of HSCR but didn’t involve in the ENS development of zebrafish. It implies that absence of intestinal ganglion cells may lead to down-regulation of *NOX5*.

## Supplementary Information

**Additional file 1.** Western blot was performed to validate the knockdown efficiency of the NOX5-MO. The results display a satisfactory knockdown power of the NOX5-MO used in our experiment. Protein in Mismatch NOX5 and Control group come from the protein mixture of 1, 3, 5 dpf in each group.

## Data Availability

The datasets generated or analyzed in this study were available from the corresponding authors on reasonable request.

## Abbreviations

HSCR: Hirschsprung Disease
GI: gastrointestinal
*NOX5*: *NADPH oxidase 5*
ENS: enteric nervous system
MO: Morpholino
