# Supplementary figures and images for: NOX5 is expressed aberrantly but not a critical pathogenetic gene in Hirschsprung disease
Source: BMC Pediatr. 2021 Mar 30;21:153. doi: 10.1186/s12887-021-02611-5 (PMC8008622; doi:10.1186/s12887-021-02611-5)

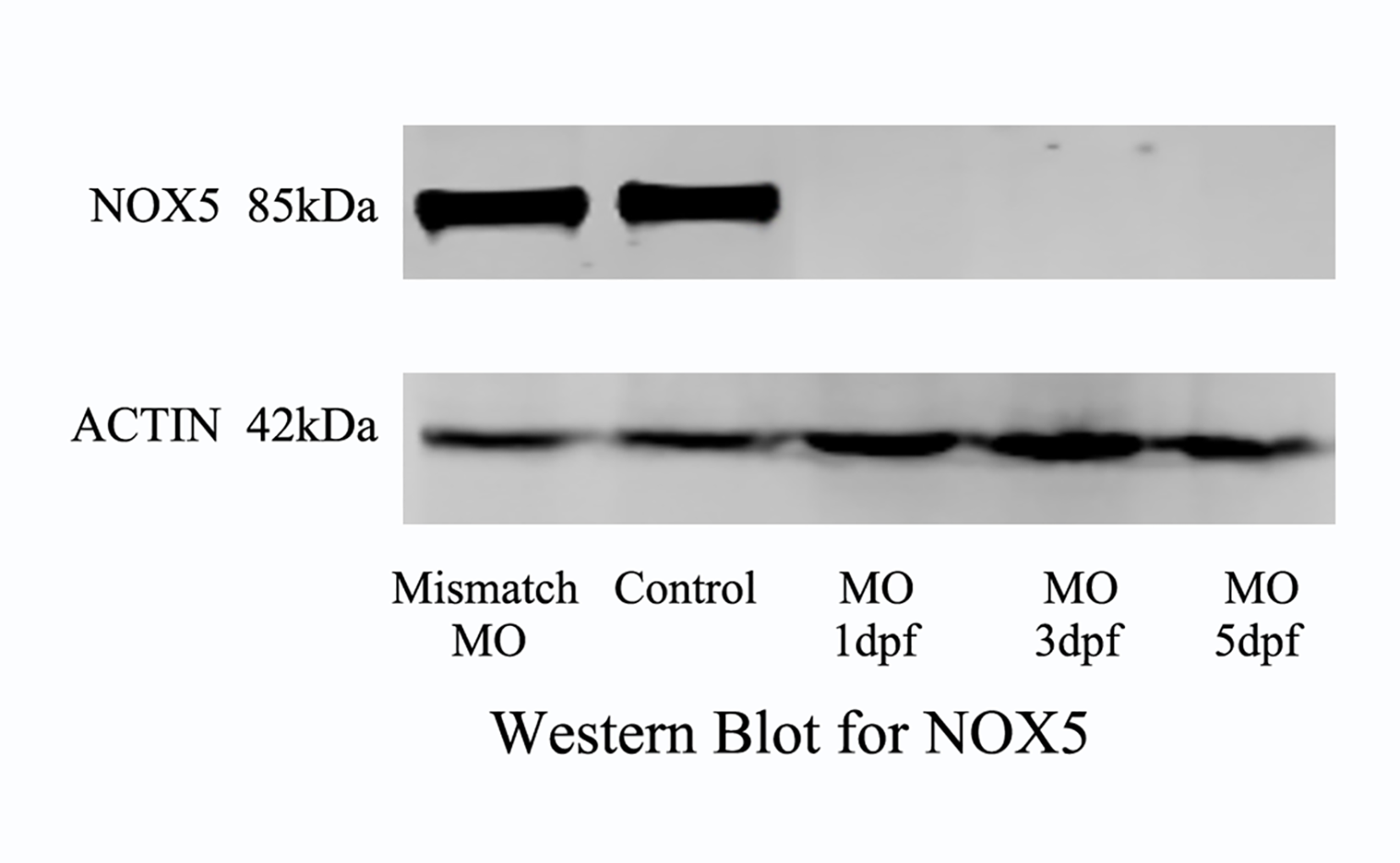

Supplement: Supplementary file 1 — Additional file 1. Western blot was performed to validate the knockdown efficiency of the NOX5-MO. The results display a satisfactory knockdown power of the NOX5-MO used in our experiment. Protein in Mismatch NOX5 and Control group come from the protein mixture of 1, 3, 5 dpf in each group. [file 12887_2021_2611_MOESM1_ESM.tif]
